# Pharmacokinetics of high-dose oral thiamine hydrochloride in healthy subjects

**DOI:** 10.1186/1472-6904-12-4

**Published:** 2012-02-04

**Authors:** Howard A Smithline, Michael Donnino, David J Greenblatt

**Affiliations:** 1 Department of Emergency Medicine, Tufts University School of Medicine and Baystate Medical Center, Springfield, MA, USA; 2 Department of Emergency Medicine, Harvard University School of Medicine and Beth Israel Deaconess Medical Center, Boston, MA, USA; 3 Program in Pharmacology and Experimental Therapeutics, Tufts University School of Medicine and Tufts Medical Center, Boston, MA, USA; 4 Baystate Medical Center, 759 Chestnut Street, Springfield, MA 01199, USA

## Abstract

**Background:**

High dose oral thiamine may have a role in treating diabetes, heart failure, and hypermetabolic states. The purpose of this study was to determine the pharmacokinetic profile of oral thiamine hydrochloride at 100 mg, 500 mg and 1500 mg doses in healthy subjects.

**Methods:**

This was a randomized, double-blind, single-dose, 4-way crossover study. Pharmacokinetic measures were calculated.

**Results:**

The AUC_0-10 hr _and Cmax values increased nonlinearly between100 mg and 1500 mg. The slope of the AUC_0-10 hr _vs dose, as well as the C_max _vs dose, plots are steepest at the lowest thiamine doses.

**Conclusion:**

Our study demonstrates that high blood levels of thiamine can be achieved rapidly with oral thiamine hydrochloride. Thiamine is absorbed by both an active and nonsaturable passive process.

**Trial Registration:**

ClinicalTrials.gov: NCT00981877

## Background

Thiamine, vitamin B_1_, was isolated in 1926 and synthesized in 1936. Its importance for preventing illness was known as early as the turn of the century. Thiamine requirements are related to energy metabolism; specifically, 0.33 mg of thiamine are required for every 4400 kJ of energy. For adults the DRI of thiamine is between 1.1 and 1.4 mg per day. The primary active form of the vitamin, thiamine diphosphate (ThDP), is also known as thiamine pyrophosphate (TPP). ThDP is a necessary cofactor for enzymes related to carbohydrate metabolism: pyruvate dehydrogenase (PDH), α-ketoglutarate dehydrogenase, and transketolase.

Thiamine, vitamin B_1_, is not synthesized by humans and is not stored in large quantities in humans [1]. One of its phosphorylated forms, thiamine diphosphate, also known as thiamine pyrophosphate, is the primary active form of the vitamin. Thiamine diphosphate is a necessary co-factor for several enzymes involved in the glycolytic pathway, citric acid cycle, pentose phosphate pathway, and degradation of branched chain amino acids.

Thiamine is used to treat various genetic disorders linked to the above metabolic pathways and thiamine deficiency syndromes (beriberi and Wernicke-Korsakoff syndrome). Oral thiamine may also have a role in treating some of the pathophysiologic conditions associated with diabetes, heart failure, and hypermetabolic states [2-5].

The optimum dosing for these beneficial effects is unknown. Rare side effects of thiamine have been attributed to allergic reactions. And although, ganglionic blockade can occur at extremely high intravenous doses, oral dosing of 3 g per day and higher have been used for extended periods of time without deleterious effects [6-8].

Free thiamine is taken-up by the body by a saturable transport system in the proximal small intestine that was thought to severely limit the amount of thiamine that can be absorbed by a single oral dose [9-11]. For this reason, alternate forms of thiamine (S-acyl thiamine derivatives and lipid-soluble thiamine disulfide derivatives), that are more absorbable by the body, had been developed [12]. However, free thiamine may be taken-up by the body by both a saturable active transport system and a nonsaturable passive process. Thus high doses of thiamine hydrochloride may be absorbable.

There is very limited pharmacokinetic data of oral thiamine at doses that are typically used and virtually no data on high-dose oral thiamine. The purpose of this study was to determine the pharmacokinetic profile of oral thiamine hydrochloride between 100 mg and 1500 mg in healthy subjects.

## Methods

### Subjects

The Institutional Review Board at Baystate Medical Center approved this study. All subjects provided written informed consent prior to participation. Fourteen healthy subjects consented to be in the study (2 dropped out on the first study day). Screening procedures included a medical history, physical exam, hematologic profile, blood chemistries, pregnancy test, and urine analysis. Subjects were not taking any medications nor were they taking any dietary or herbal supplements.

### Study design and procedures

Subjects participated in a randomized, double-blind, single-dose, 4-way crossover study with a minimum of 1 week elapsing between trials. The 4 treatment groups were:

1. Placebo

2. 100 mg thiamine hydrochloride

3. 500 mg thiamine hydrochloride

4. 1500 mg thiamine hydrochloride

Subjects fasted overnight except for water. In the morning they had a blood sample drawn followed by a standardized breakfast. After 1 hour they were administered the study medication. Additional blood specimens were obtained immediately prior to the study medication and at 0.5, 1, 1.5, 2, 3, 4, 5, 6, 8, and 10 hours after taking the study medication. Subjects received a standardized lunch after the 6-hour blood draw. All blood specimens were drawn in duplicate (for plasma and whole blood assays) in 3 mL lavender-top vacutainers^® ^containing K_2_EDTA. They were immediately placed in an ice bath and protected from the light. One vacutainer^® ^of each pair was centrifuged at 3000 RPM for 20 min at 4°C to separate the plasma. The plasma sample and whole blood sample were then frozen at -70°C.

### Study medication

Thiamine tablets each containing 100 mg of thiamine hydrochloride were used (Amneal Pharmaceuticals). These were placed intact into opaque capsules. Identical capsules containing sucrose tablets were used as placebo such that the same number of capsules were used for each trial.

### Analysis of blood specimens

Quest Diagnostics analyzed all specimens for total thiamine using HPLC [4]. At Quest Diagnostics, plasma was deproteinized and then incubated with acid phosphatase to convert thiamine phosphate esters to free thiamine. The free thiamine was then oxidized to thiochrome by the addition of alkaline potassium ferricyanide. Depending on the age of the column and the temperature of the room, thiochrome retention time varied from 2.5 to 3.0 min. The mixture was injected to a Supelco (Bellefonte, PA, USA) high-performance liquid chromatographic column (7.5 cm^3 ^4.6 mm, particle size 3 mm) connected to a high-performance liquid chromatographic system using a Hitachi (Pleasant, CA, USA) pump, autosampler, and fluorescent detector (excitation wavelength 365 nm, emission wavelength 440 nm). The mobile phase was 75 mmol/L of potassium phosphate at pH 7.5 with 25% methanol. The flow rate was set at 1.0 mL/min. Through this process, the thiochrome was then separated from other interfering substances and then measured fluorometrically. The amount of total thiamine in an unknown sample is proportional to the amount of thiochrome formed. The limit of quantification is 7 nmol/L. The assay range is 7 to 450 nmol/L. Samples with values above this range were diluted. The coefficient of variation for both the plasma and whole blood thiamine assays was calculated from the data collected in this study.

### Pharmacokinetic and statistical analysis

The thiamine levels were corrected by subtracting the baseline value. The baseline value was calculated as the average of the -1-hour and 0-hour values. Net systemic exposure to thiamine in each subject in each trial was quantitated using the area under the whole blood or plasma concentration curve from time zero through 10 hours after dosage (AUC_0-10 hr_). This was calculated using the cubic splines method. The overall effect of treatment condition (thiamine dose) on AUC_0-10 hr _in blood and plasma was tested using analysis of variance (ANOVA) for repeated measures. This was followed by the Student-Newman-Keuls procedure, nonparametric form, for evaluating all pairwise comparisons of the mean AUC_0-10 hr _values for the dosage groups. The peak thiamine concentration (C_max_) and the time to peak thiamine concentration (T_max_) were also calculated. Finally, half-life (t_1\2_) values were calculated where the terminal phase appeared log-linear.

The relative impact of active and passive absorption were assessed by comparing the AUC_0-10 hr _vs dose for 0 mg to 100 mg, 100 mg to 500 mg, and 500 mg to 1500 mg doses using multilevel mixed-effects linear regression. This was then repeated for C_max _vs dose.

The data from the placebo trials was used to calculate the coefficient of variation for both the plasma and whole blood assays using the logarithmic method. The coefficient of variation for plasma was 0.15 and the coefficient of variation for whole blood was 0.11.

Stata statistical software version 11 was used for all calculations.

## Results

Fourteen subjects consented to be in the study. Of these, 2 dropped out because of poor venous access during the 1^st ^trial and their data are not included in this analysis. Table 1 lists the demographics of the subjects. Table 2 shows the pharmacokinetic values for the whole blood and plasma thiamine measures.

**Table 1:** Demographics

Age (years)	29 (10)
Weight (kg)	87 (20)

Gender	

Female	64%

Male	36%

Race	

White	79%

Black	21%

Ethnicity	

Hispanic	36%

Non-Hispanic	64%

Hemoglobin (g/dL)	15 (4)

Sodium (mmol/L)	139 (2)

Potassium (mmol/L)	4.1 (0.3)

Chloride (mmol/L)	102 (2)

Bicarbonate (mmol/L)	27 (2)

Glucose (mg/dL)	92 (9)

BUN (mg/dL)	13 (3)

Creatinine (mg/dL)	0.8 (0.2)

values are either mean (SD) or percentages

**Table 2:** Pharmacokinetic Values

PARAMETER	THIAMINE DOSE		
	**100 mg**	**500 mg**	**1500 mg**

AUC_0-10 hr _(nmol/Liter × hours)			

whole blood	214 ± 69	623 ± 178	2046 ± 1222

plasma	177 ± 62	612 ± 257	2059 ± 1415

C_max _(nmol/Liter)			

whole blood	40 ± 11	95 ± 27	385 ± 188

plasma	39 ± 13	113 ± 42	397 ± 250

T_max _(hours)			

whole blood	3.43 ± 1.69	4.14 ± 1.57	4.14 ± 0.90

plasma	3.14 ± 1.05	3.18 ± 0.98	4.27 ± 1.01

All values are presented as mean ± standard deviation. p < 0.05 for all pairwise comparisons (plasma and whole blood values were analyzed separately)

**Figure 1:**
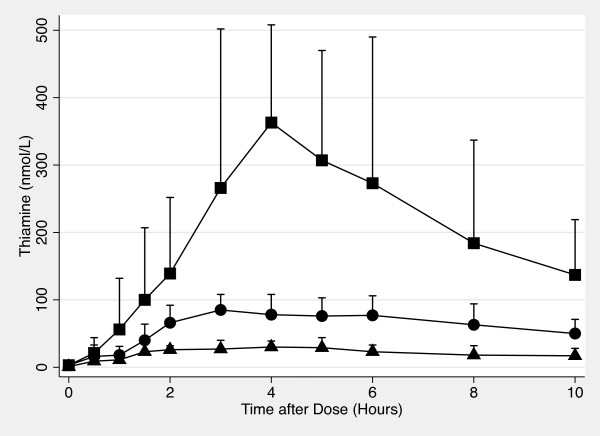
**Oral Thiamine Whole Blood Concentration vs Time Plot**. The concentration of thiamine in plasma from 0 hour to 10 hours after 100 mg (♦), 500 mg (●) and 1500 mg (■) of oral thiamine. Error bars are standard deviations.

**Figure 2:**
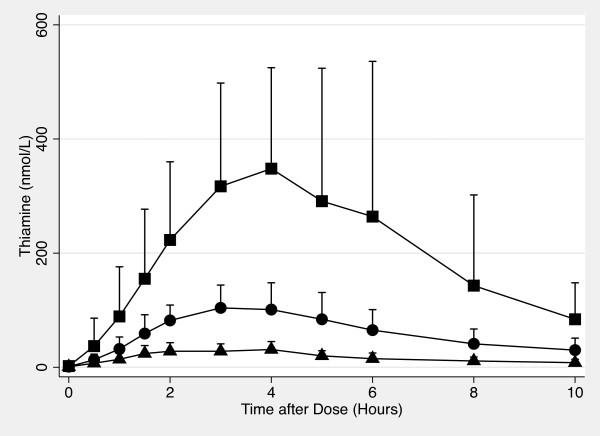
**Oral Thiamine Plasma Concentration vs Time Plot**. The concentration of thiamine in whole blood from 0 hour to 10 hours after 100 mg (♦), 500 mg (●) and 1500 mg (■) of oral thiamine. Error bars are standard deviations.

Figures 1 and 2 show whole blood and plasma thiamine concentrations vs time plots for each thiamine dose. The overall effect of dose was significant (Plasma: ANOVA *p *< 0.001; Whole Blood: ANOVA *p *< 0.001). Additionally, the mean AUC_0-10 hr _for each dose was also significantly different from the others (Student-Newman-Keuls procedure, *p *< 0.05).

**Figure 3:**
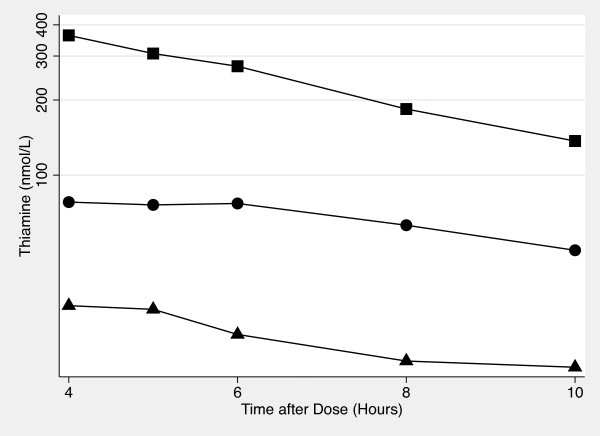
**Semi-log Plot of the Terminal Phase of Oral Thiamine Whole Blood Concentration vs Time**. The concentration of thiamine in whole blood from 4 hour to 10 hours after 100 mg (♦), 500 mg (●) and 1500 mg (■) of oral thiamine.

**Figure 4:**
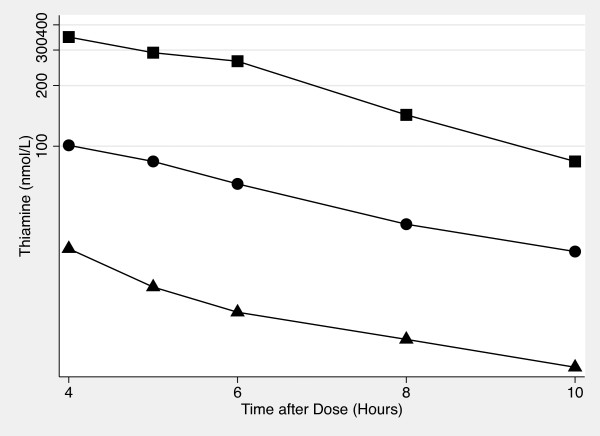
**Semi-log Plot of the Terminal Phase of Oral Thiamine Plasma Concentration vs Time**. The concentration of thiamine in plasma from 4 hour to 10 hours after 100 mg (♦), 500 mg (●) and 1500 mg (■) of oral thiamine.

Figures 3 and 4 show semi-log plots of the terminal phase of thiamine concentration vs time plots for each thiamine dose for whole blood and plasma. The plots suggest that the terminal phase of thiamine concentration vs time for the 1500 mg dose (whole blood) as well as the 500 mg and 1500 mg doses (plasma) are log-linear. The half-life was calculated for these doses: 4.78 ± 2.02 hrs (1500 mg, whole blood), 3.92 ± 2.24 hrs (500 mg, plasma), and 2.97 ± 1.05 hrs (1500 mg, plasma).

**Figure 5:**
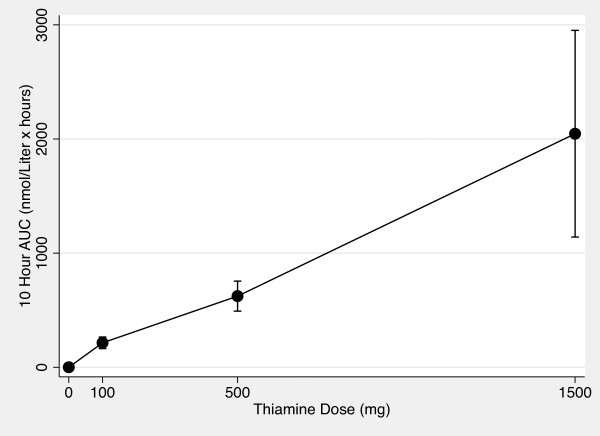
**Whole Blood 0 to 10 Hour AUC vs Thiamine Dose Plot**. The mean area under the curve values for whole blood thiamine measures from time 0 hour to time 10 hours vs thiamine dose after 0 mg, 100 mg, 500 mg and 1500 mg of oral thiamine. Error bars are 95% confidence intervals.

**Figure 6:**
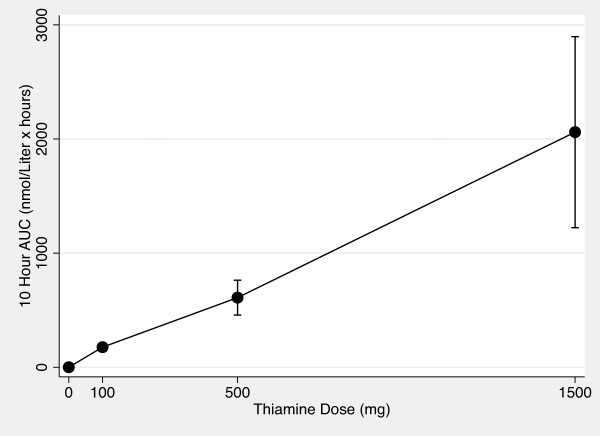
**Plasma 0 to 10 Hour AUC vs Thiamine Dose Plot**. The mean area under the curve values for plasma thiamine measures from time 0 hour to time 10 hours vs thiamine dose after 0 mg, 100 mg, 500 mg and 1500 mg of oral thiamine. Error bars are 95% confidence intervals.

**Figure 7:**
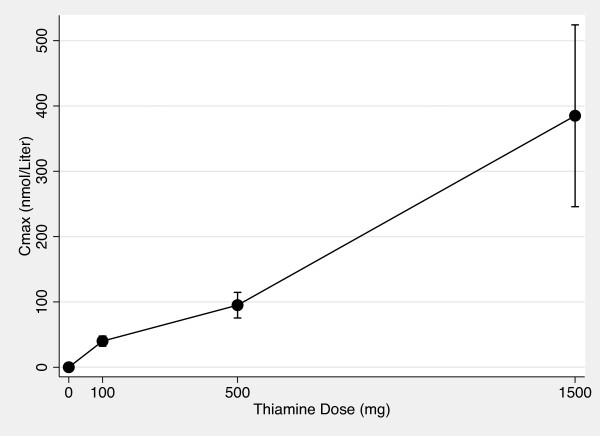
**Whole Blood C_max _vs Thiamine Dose Plot**. The mean maximum whole blood thiamine concentration between time 0 hour and time 10 hours vs thiamine dose after 0 mg, 100 mg, 500 mg and 1500 mg of oral thiamine. Error bars are 95% confidence intervals.

**Figure 8:**
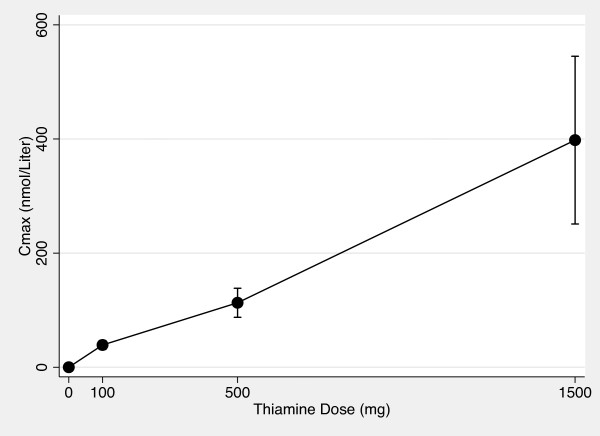
**Plasma C_max _vs Thiamine Dose Plot**. The mean maximum whole blood thiamine concentration between time 0 hour and time 10 hours vs thiamine dose after 0 mg, 100 mg, 500 mg and 1500 mg of oral thiamine. Error bars are 95% confidence intervals.

Figures 5 and 6 show the AUC_0-10 hr _vs thiamine dose plots for whole blood and plasma. Figures 7 and 8 show the C_max _vs thiamine dose plots for whole blood and plasma. These plots suggest that the slope is steepest between 0 mg and 100 mg doses of thiamine. This was then tested in separate multilevel mixed effects models and the results are shown in Table 3. The steepest slope is between 0 mg and 100 mg suggesting that while active and passive transport probably occur at all doses, active and transport is more dominant at lower thiamine doses compared to higher thiamine doses. The lack of reaching statistical significance comparing the 0 mg - 100 mg to the 500 mg - 1500 mg segment should be interpreted cautiously because of the small sample size of this study.

**Table 3:** Comparison of Slopes for 0 to 10 Hour AUC vs Thiamine Dose

	AUC_0-10 hr_		C_max_	
**Segment**	**Slope**	*p*-value*	**Slope**	*p*-value*

0 to 100 mg				

whole blood	2.14		0.40	

plasma	1.76		0.39	

100 mg to 500 mg				

whole blood	1.02	< 0.01	0.14	< 0.01

plasma	1.09	0.06	0.18	< 0.01

500 mg to 1500 mg				

whole blood	1.42	0.06	0.29	0.03

plasma	1.45	0.40	0.29	0.08

*Compared to the 0 to 100 mg segment

## Discussion

The mechanism of how thiamine is absorbed has been somewhat controversial. One group of researchers concluded that thiamine is only absorbed by a saturable active transport mechanism in the proximal small intestine; however other researchers have shown that thiamine is also absorbed by a passive process [13]. Several studies by Thomson demonstrated that a maximum amount between 4.8 and 8.3 mg of thiamine could be absorbed by a single oral dose of thiamine hydrochloride. In these studies, subjects were given a single dose oral thiamine between 1 and 20 mg [9-11]. In another study, Morrison, found that very little thiamine was excreted in urine when doses above 2.5 mg were given orally [14]. This saturable active transport mechanism has been attributed to two carriers, thiamine transporter-1 and thiamine transporter-2.

Using a study design where thiamine was infused directly into the lumen of the small intestine, Hoyumpa demonstrated that thiamine was absorbed by an active process at low concentrations (0.2 to 2.0 μM) and by a passive process at higher concentrations (5.0 to 50.0 μM) [15-18]. This was also found in an in vitro study [19]. Thomson found a linear relationship between urinary excretion and oral dose of thiamine between 10 and 50 mg [20]. This is further supported by Weber who gave a single dose of oral thiamine to 3 subjects (50, 100, or 200 mg) and found that the subject given the largest oral dose also had the highest plasma thiamine levels [21]. Although, the design of this study and its small sample size limits any conclusions.

Studies to determine the optimal dosing of thiamine for various conditions have not been performed and dosing recommendations appear to be based on limited data. Thiamine deficiency syndromes are typically initially treated with intravenous thiamine between 100 mg once a day and 500 mg three times a day [22,23]. Oral dosing of thiamine up to 100 mg/kg divided three times a day have been reported to be required to treat children with genetic abnormalities of pyruvate dehydrogenase. Children with these abnormalities who were not improved by taking thiamine, may have been treated with an inadequate dosage [24]. In studies of Alzheimer's disease, subjects were treated with 1000 mg of oral thiamine hydrochloride three times a day for 2 to 12 months without any reports of adverse effects [7,25,26]. In a separate experiment, subjects were titrated up to 8000 mg per day over a 1-year period. The only side effects reported were nausea and indigestion in 2 subjects when they reached 7000 and 7500 mg per day [7]. There have been several clinical trials of thiamine derivatives for a variety of disorders that used doses between 300 and 900 mg per day in divided doses for periods up to 3 months. No side effects were reported in these studies [2,27-29].

Alternate forms of oral thiamine (S-acyl thiamine derivatives and lipid-soluble thiamine disulfide derivatives) have been developed because they have a much higher bioavailability than thiamine hydrochloride [12]. Thiamine hydrochloride has been estimated to have a bioavailability between 3.7% and 5.3% [21,30]. However, it is not clear that these thiamine derivatives are needed. First, tissue uptake is highly variable across different tissues and different derivatives [12]. Second, oral thiamine hydrochloride when given over a 1-week period produce blood levels that approach those obtained by intramuscular and intravenous administration [31,32]. Finally, in vitro studies that have compared thiamine to thiamine derivatives have generally found them to have similar effects [33-38]. In studies where a thiamine derivative was thought to be superior to thiamine hydrochloride, the difference could be completely explained by differences in bioavailability [39-41].

This study has demonstrated that the absorption mechanism is not saturable up to 1500 mg. Our results are consistent with a combination of passive and active transport. The active transport plays a larger role in thiamine absorption at lower doses compared to higher doses. These results contradict the results found by Thomson but is consistent with the animal studies done by Hoyumpa. The constant difference between plasma and whole blood thiamine levels is consistent with a rapid equilibrium between red blood cells and plasma.

This study was limited in that we did not measure tissue levels of thiamine nor did we measure biological effects of high dose oral thiamine hydrochloride. Additionally, while no side effects were reported, this study was not designed to detect adverse events. Our calculation of the coefficient of variation has additional limitations. It includes error related to the analytic method in addition to error related to biologic variation as well as all of the steps between obtaining the blood and its preparation prior to analysis. A greater understanding of the absorption process could have determined if we studied a larger number of thiamine doses and followed the subjects for a longer period of time.

## Conclusions

In conclusion, our study demonstrates that high blood levels of thiamine can be achieved rapidly with oral thiamine hydrochloride. Thiamine is absorbed by both an active and unsaturable passive transport mechanism up to 1500 mg.

## Competing interests

The authors declare that they have no competing interests.

## Authors' contributions

HAS made substantial contributions to conception and design, acquisition of data, analysis and interpretation of data, drafting the manuscript. MD made substantial contributions to analysis and interpretation of data and revised the manuscript critically for important intellectual content. DJG made substantial contributions to conception and design, analysis and interpretation of data, and revised the manuscript critically for important intellectual content. All authors read and approved the final manuscript.

## Pre-publication history

The pre-publication history for this paper can be accessed here:

http://www.biomedcentral.com/1472-6904/12/4/prepub
